# On an ensemble algorithm for clustering cancer patient data

**DOI:** 10.1186/1752-0509-7-S4-S9

**Published:** 2013-10-23

**Authors:** Ran Qi, Dengyuan Wu, Li Sheng, Donald Henson, Arnold Schwartz, Eric Xu, Kai Xing, Dechang Chen

**Affiliations:** 1 Computer Science and Electrical Engineering, University of Maryland at Baltimore County, Baltimore, MD 21250, USA; 2 Department of Computer Science, The George Washington University, Washington, DC 20052, USA; 3 Department of Mathematics, Drexel University, Philadelphia, PA 19104, USA; 4 Division of Cancer Control and Epidemiology, The George Washington University Cancer Institute, Washington, DC 20037, USA; 5 Department of Pathology, The George Washington University Medical Center, Washington, DC 20037, USA; 6 Department of Biology, University of Virginia, Charlottesville, VA 22904, USA; 7 Department of Computer Science, University of Science and Technology of China, P. R. China; 8 Department of Preventive Medicine and Biometrics, The Uniformed Services University of The Health Sciences, MD 20814, USA

## Abstract

**Background:**

The TNM staging system is based on three anatomic prognostic factors: Tumor, Lymph Node and Metastasis. However, cancer is no longer considered an anatomic disease. Therefore, the TNM should be expanded to accommodate new prognostic factors in order to increase the accuracy of estimating cancer patient outcome. The ensemble algorithm for clustering cancer data (EACCD) by Chen *et al*. reflects an effort to expand the TNM without changing its basic definitions. Though results on using EACCD have been reported, there has been no study on the analysis of the algorithm. In this report, we examine various aspects of EACCD using a large breast cancer patient dataset. We compared the output of EACCD with the corresponding survival curves, investigated the effect of different settings in EACCD, and compared EACCD with alternative clustering approaches.

**Results:**

Using the basic *T *and *N *definitions, EACCD generated a dendrogram that shows a graphic relationship among the survival curves of the breast cancer patients. The dendrograms from EACCD are robust for large values of *m *(the number of runs in the learning step). When *m *is large, the dendrograms depend on the linkage functions.

The statistical tests, however, employed in the learning step have minimal effect on the dendrogram for large *m*. In addition, if omitting the step for learning dissimilarity in EACCD, the resulting approaches can have a degraded performance. Furthermore, clustering only based on prognostic factors could generate misleading dendrograms, and direct use of partitioning techniques could lead to misleading assignments to clusters.

**Conclusions:**

When only the Partitioning Around Medoids (PAM) algorithm is involved in the step of learning dissimilarity, large values of *m *are required to obtain robust dendrograms, and for a large *m *EACCD can effectively cluster cancer patient data.

## Background

Accurate outcome (survival) estimation is often the key in the successful treatment of cancer patients. Estimation depends on clinical or laboratory variables or factors that are linked to patient outcome. Found in all specialties of medicine, predictive factors take on significant clinical meaning when treatment options are available, but they become more important if treatment options are limited and not always effective.

Currently, the most common predictive factors in cancer medicine are the three variables *T , N*, and *M *of the TNM (*T*umor, Lymph *N*ode, and *M*etastasis) staging system that define the anatomic extent of disease [1]. The "*T*" usually refers to the size of the primary tumor, "*N*" refers to the presence or absence of metastatic deposits in regional lymph nodes, and "*M*" indicates the presence of metastatic disease. With the TNM staging system, levels of these three variables are combined, and patients are classified into four stage groups according to different combinations of the levels. Then the outcome estimation of patients is based on the survival function estimated for each stage.

The TNM was created by surgeons primarily for surgery. However, cancer medicine no longer lives in the world where surgery remains the only treatment. The field of cancer is now characterized by screening and early detection, proteogenomics, multiple therapies, and a bewildering array of prognostic factors. Advances in molecular medicine, imaging, and therapeutics are now forcing us to integrate additional prognostic factors for more accurate estimation of patient outcome [2-5]. Therefore, to improve the estimation of outcome, methods are needed to incorporate additional prognostic factors into the TNM without changing the anatomic definitions.

The ensemble algorithm for clustering cancer data (EACCD) by Chen *et al. *[6] is designed to explore expansion of the TNM by integrating additional factors into the system. Though many results on using EACCD have been reported, there has been no study available to analyze the algorithm. In this report, we present an analysis of EACCD by using a large breast cancer dataset. We compared the output of EACCD with the corresponding survival curves, investigated the effect of different settings for EACCD, and compared EACCD with several other clustering approaches. This report represents an extensive expansion of the work in [7].

## Method

### EACCD

In this section, we describe the EACCD. Our presentation allows a collection of partition methods in constructing dissimilarities and thus is more general than that in [6]. Let the record for the *i*th patient be (*x_i0_,x_i1_*,...,*x_ip_,δ_i_*), where *x_i0 _*equals the observed time (censored or un-censored survival time), *x_ij _*are measurements on variables (factors) *X_j _*for *j *= 1, ... , *p*, and *δ_j _*is the event indicator which is defined to be 1 if the event (e.g., death) has occurred and 0 if the time on study is right-censored. Define a combination to be a set of(*x_i0_,x_i1_*,...,*x_ip_,δ_i_*) that corresponds to one level of each variable (A continuous variable should be discretized). EACCD is an algorithm used to cluster combinations. In the algorithm, dissimilarity between two combinations is learnt by repeatedly using some clustering (partitioning) approaches based on criterion minimization, and then the learnt dissimilarity measure is used with a hierarchical clustering method in order to find final clusters of combinations. The algorithm involves the following three steps.

### Computing initial dissimilarity

Assume that there are a total of *n *combinations **x**_1_, **x**_2_, ... , **x***_n_*. Then the following initial dissimilarity measure $dis_{0}\left(x_{i},x_{i^{\prime }}\right)$ is defined between two combinations **x***_i _*and **x***_i'_*:

(1) $$dis_{0}\left({\text{x}}_{i},{\text{x}}_{i^{\prime }}\right)=d_{0}.$$

Here *d_0 _*is the value of a test statistic (e.g., the log-rank test statistic [8]) used to determine if three is a difference in the survival functions between the two populations associated with **x***_i _*and **x***_i'_*. In general, $dis_{0}\left(x_{i},x_{i^{\prime }}\right)$ assumes any non-negative value.

### Computing learnt dissimilarity

Let *C *denote a cluster assignment, assigning the *i*th combination to a cluster, i.e., *C*(**x***_i_*) ∈ ( {1, 2, ... ,*K*} for a predetermined integer *K*. The optimal assignment C* is obtained by minimizing the "within-cluster" scatter, i.e., by solving the following discrete optimization problem:

(2) $$\underset{C,{\left\{i_{k}\right\}}_{1}^{K}}{\text{min}}\overset{K}{\underset{k=1}{\sum }}\overset{}{\underset{C\left(x_{i}\right)=k}{\sum }}dis_{0}\left(x_{i},x_{i_{k}}\right).$$

Numerical procedures (e.g., the Partitioning Around Medoids (PAM) [9]) are employed to find the solution to the above optimization problem. For the data {x_1_, x_2_, ... , x*_n_*}, one *K *and one clustering or partitioning method may be chosen to partition the data into *K *clusters. However, the final assignment usually depends on the selected method and the initial reallocation. To overcome this, one can run this partition process *m *times. Each time a number *K *is randomly picked from a given interval [*K*_1_,*K*_2_] and a partitioning procedure is also randomly selected. Define *δ_l_*(*i, j*) = 1 if the *l*th run of a procedure does not assign x*_i _*and x*_j _*into the same cluster; and *δ_l_*(*i, j*) = 0 otherwise. And then define the following dissimilarity measure between two combinations **x***_i _*and **x***_j_*:

(3) $$dis\left(x_{i}\text{,}x_{j}\right)=\frac{\overset{N}{\underset{l=1}{\sum }}\delta_{l}\left(i,j\right)}{m}.$$

Note that *dis*(**x***_i_*, **x***_j_*) ranges from 0 to 1. A smaller value of *dis*(**x***_i_*, **x***_j_*) indicates that **x***_i _*and **x***_j _*most likely come from the same "hidden" group. In other words, a smaller dissimilarity *dis*(**x***_i_*, **x***_j_*) is expected to imply a smaller difference between the two survival functions associated with the two combinations.

### Hierarchical clustering

This step clusters the combinations by applying a linkage method [10] and the learnt dissimilarity *dis*(**x***_i_*, **x***_j_*). The primary output of EACCD is a dendrogram that provides a summary of the survival experiences based on the levels of prognostic factors, and thus has multiple applications.

The algorithm is outlined in Algorithm 1. Note that if only PAM is used for computing the learnt dissimilarity, then the algorithm reduces to that introduced in [6].

### Data set

A breast cancer patient dataset was obtained from the Surveillance, Epidemiology, and End Results (SEER) Program of the National Cancer Institute [11]. Because of its size, quality control, broad US representation, unbiased ascertainment, and 35-year history, the Program is ideal for evaluating algorithms. We selected data for breast cancer from the years 1990-2000 using SEER's Case Listing. During the selection process, we followed the definitions for tumor size and number of involved lymph nodes as published by the American Joint Committee on Cancer [1]. The dataset contained 202, 219 cases having complete records on *T *(tumor size), *N *(nodal status), *X *(survival time), and *δ *(censoring status). The factors *T *and *N *have 3 and 4 categories, respectively, as listed in Table 1. Therefore there are 12(3 × 4) combinations based on *T *and *N*. And for convenience, we denoted by *T*1*N*0 the combination formed using categories *T*1 and *N*0, by *T*1*N*1 the combination formed using categories *T*1 and *N*1, and so on.

**Table 1:** Definitions of *T*and *N *for SEER breast cancer cases from 1990-2000.

Prognostic factors	Categories	Level
Tumor size	*T*1(*T ≤ *2cm) *T*2(2cm *< T ≤ *5cm ) *T*3(*T >*5cm)	1 2 3
Nodal status	*N*0(No positive axillary nodes) *N*1(1 - 3 nodes contain tumor) *N*2(4 - 10 nodes contain tumor) *N*3(More than 10 nodes contain tumor)	1 2 3 4

**Algorithm 1 **Ensemble algorithm for clustering cancer patient data

1. Define the initial dissimilarity *dis*_0 _in (1).

2. Obtain a collection of procedures for solving (2). Choose *m, K*_1_, and *K*_2_, and run these procedures *m *times, where for each time, a procedure is randomly selected from the collection and a *K *is randomly chosen from the interval [*K*_1_, *K*_2_]. Then construct the pairwise dissimilarity measure *dis *by using the equation (3).

3. Cluster the combinations by applying a linkage method and the learnt measure *dis*.

### Evaluation of EACCD

We evaluated EACCD by performing a series of experiments using the programming language "R" [12]. The PAM algorithm was used in the second step of EACCD throughout the evaluation. Random medoids were initially selected for the PAM in all cases except for A_4_, described below, where the default initial medoids in "R" were used.

The evaluation began with the application of the algorithm to clustering the breast cancer patients. We examined how the algorithm grouped the patients and compared this grouping with the possible grouping pattern exhibited in the survival curve plot. For the experiments, the log-rank test statistic [8] was used to determine the initial dissimilarity in the first step of the algorithm. In the second step we chose *K*_1 _= 2, *K*_2 _= 11 (the total number of combinations minus one). The PAM algorithm was repeatedly executed for *m *= 10000 times. In the third step, the average linkage hierarchical clustering technique [10] was used.

We then examined the effect of different settings in EACCD on the dendrogram generated by the algorithm. There were mainly three "factors" that could influence the final result in EACCD: test (the statistical test employed in determining the initial dissimilarity in Step 1 of the algorithm), *m *(the number of rounds of partitioning procedures performed in obtaining the learnt dissimilarity in Step 2) and the linkage function (the linkage function used in the hierarchical clustering procedure in Step 3). The effects of these "factors" were analyzed by varying their "values." While the value of *m *was chosen from {10, 20, 50, 100, 500, 1000, 5000, 10000, 20000, 30000}, we considered three tests (the log-rank test, the Gehan-Wilcoxon's test, and the Tarone and Ware's test [8]) and three linkage functions (the average linkage, the complete linkage, and the single linkage [10]).

Finally, we compared EACCD with four additional approaches that could be used to cluster the cancer patient data. These approaches were either straight forward or modifications of EACCD. Specifically the four approaches *A*_1_,*A*_2_,*A*_3_,*A*_4 _are described below. For demonstration, we used *m *= 10000, the log-rank test, and the average linkage for the setting of EACCD.

#### Approach A_1_

This was tailored from the EACCD, omitting the learning step for dissimilarity. The initial dissimilarity measure *dis*_0 _in (1) was obtained first using the log-rank test and then standardized into 0[1] by the equation $dis_{A_{1}}^{S}=dis_{0}/max\;\left\{dis_{0}\right\}$. The standardized initial dissimilarity values were then used in the hierarchical clustering procedure with the average linkage function.

#### Approach A_2_

In testing the differences between two survival curves associated with two combinations, a smaller p-value normally indicates a larger difference between the survival curves. Therefore, 1 − *p*, ranging from 0 to 1, could be used as the pairwise dissimilarity measure between two combinations in light of the survival. In the approach of *A*_2_, this dissimilarity 1 − *p*, from the log-rank test, was directly used in the hierarchical clustering procedure with the average linkage function. The learning step for dissimilarity was not required.

#### Approach A_3_

In *A*_3_, we considered one traditional procedure in clustering the cancer data by using the two factors *T *and *N*. For each combination, let $\hat{T}$ denote the average value of *T *and $\hat{N}$ the average value of *N*. We could use $\hat{T}$ and $\hat{N}$ to represent the *T *and *N *value of the combination, respectively. Since $\hat{T}$ has a much larger range than $\hat{N},$ a linear transformation was performed to standardize $\hat{T}$ and $\hat{N}$ into 0[1] as ${\hat{T}}^{s}$ = ($\hat{T}$ − min{$\hat{T}$})/(max{$\hat{T}$}− min{$\hat{T}$}) and ${\hat{N}}^{s}$ = ($\hat{N}$ − min{$\hat{N}$})/(max{$\hat{N}$}− min{$\hat{N}$}). Let ${\hat{T}}_{i}^{s}$ and ${\hat{N}}_{i}^{s}$ be the standardized values for combination x*_i_*. Then the dissimilarity between combinations x_i _and x_j _was defined as *dis*(x*_i_*, x*_j_*)$=|{\hat{T}}_{i}^{s}={\hat{T}}_{j}^{s}|+{\hat{N}}_{i}^{s}-{\hat{N}}_{j}^{s}|.$ This dissimilarity *dis *was then standardized into the range of 0[1] using $dis_{A_{3}}^{s}=dis/\text{max}\left\{dis\right\}$. Based on $dis_{A_{3}}^{s}$, hierarchical clustering with the average linkage was then performed.

#### Approach A_4_

In *A*_4 _the PAM clustering algorithm was directly used to partition the cancer data. The quantity $dis_{A_{1}}^{s}$ in the approach *A*_1 _was taken as the input dissimilarity measurement. The number of clusters was set at 2, ... , 11, respectively, and thus 10 partition results were available.

## Results and discussion

### An application study

**Figure 1:**
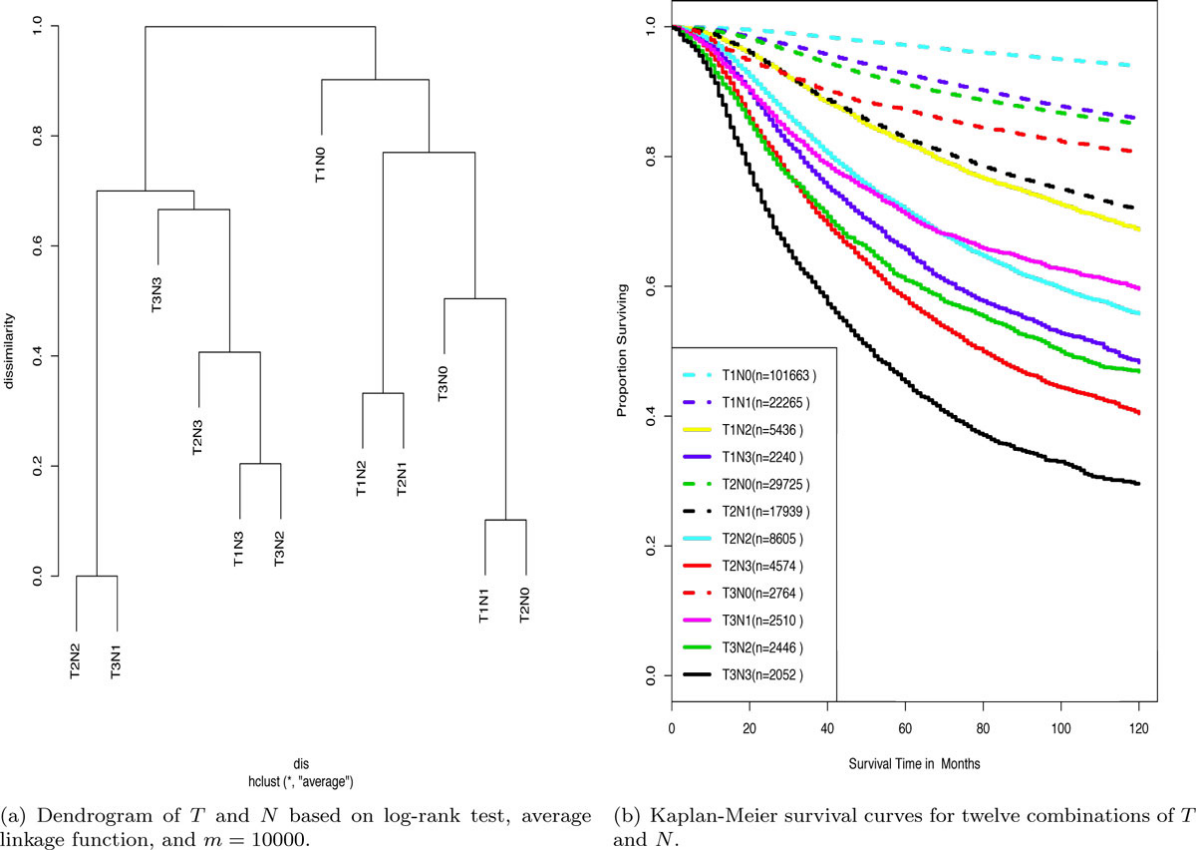
**Dendrogram of *T *and *N *from EACCD and survival curves for *T *and *N *combinations**.

EACCD, when applied to the breast cancer data, generated a dendrogram (Figure 1(a)) that exhibits one relationship among 12 survival curves corresponding to the 12 combinations.

More specifically, the dendrogram provided an overall view of the relationship among the outcomes as the levels of prognostic factors were changed. We begin with the leftmost side or branch of Figure 1(a). The dissimilarity (difference) between the survival curve of *T*1*N*3 and the survival curve of *T*3*N*2 is 0.20. Merge *T*1*N*3 with *T*3*N*2 and denote by *T*1*N*3 + *T*3*N*2 the resulting group of patients. Then the difference between the survival curve of *T*1*N*3 + *T*3*N*2 and the survival curve of *T*2*N*3 is 0.41. Merge *T*1*N*3 + *T*3*N*2 with *T*2*N*3 and denote the resulting group of patients by *T*1*N*3 + *T*3*N*2 + *T*2*N*3. Then in light of survival, this group *T*1*N*3 + *T*3*N*2 + *T*2*N*3 differs from *T*3*N*3 by a value of 0.67. Merging *T*3*N*3 with *T*1*N*3 + *T*3*N*2 + *T*2*N*3 and denoting the resulting group by *T*1*N*3 + *T*3*N*2 + *T*2*N*3 + *T*3*N*3, then *T*2*N*2 + *T*3*N*1 differs from *T*1*N*3 + *T*3*N*2 + *T*2*N*3 + *T*3*N*3 by a value of 0.70 in terms of survival. Here *T*2*N*2 + *T*3*N*1 is the group from merging *T*2*N*2 with *T*3*N*1, where *T*2*N*2 differs from *T*3*N*1 by a value of 0.00. Denote by *T*1*N*3 + *T*3*N*2 + *T*2*N*3 + *T*3*N*3 + *T*2*N*2 + *T*3*N*1 the result from merging *T*2*N*2 + *T*3*N*1 and *T*1*N*3 + *T*3*N*2 + *T*2*N*3 + *T*3*N*3. The above shows the relationship among the survival curves of the combinations contained in the left branch of the dendrogram. A similar interpretation applies to the survival curves of the combinations in the right branch of the dendrogram. Finally, the left branch differs from the right branch by a value of 1.0 in light of survival. That is, 1.0 is the difference between the survival curve of the group *T*1*N*1 + *T*2*N*0 + *T*3*N*0 + *T*1*N*2 + *T*2*N*1 + *T*1*N*0 and the survival curve of the group *T*1*N*3 + *T*3*N*2 + *T*2*N*3 + *T*3*N*3 + *T*2*N*2 + *T*3*N*1.

The relationship among the survival curves exhibited in the dendrogram of *T *and *N *(Figure 1(a) ) can be confirmed by visually checking the 12 survival curves shown in Figure 1(b). These survival curves were constructed by the Kaplan-Meier procedure [8]. The survival curves in Figure 1(b) can be divided into two groups, group 1 consisting of the lower six curves and group 2 consisting of the upper six curves. The curves in group 1 and group 2 appear on the left and right branches in Figure 1(a), respectively of the dendrogram. Thus, from a practical perspective, the dendrogram initially divides the patients into those with a favorable outcome and those with an unfavorable outcome. A visual check of group 1 in Figure 1(b) shows certain differences among the curves. For instance, the two closest curves are the curve of *T*2*N*2 and the curve of *T*3*N*1, and the next two closest curves are the curves of *T*1*N*3 and *T*3*N*2. If we merge combinations in the order of increasing differences between survival rates, we would first merge *T*2*N*2 with *T*3*N*1, and then merge *T*1*N*3 with *T*3*N*2, merge *T*1*N*3 + *T*3*N*2 with *T*2*N*3, merge *T*1*N*3 + *T*3*N*2 + *T*2*N*3 with *T*3*N*3, and finally, merge *T*1*N*3 + *T*3*N*2 + *T*2*N*3 + *T*3*N*3 with *T*2*N*2 + *T*3*N*1. Clearly, this observation coincides with the relationship among survival curves depicted by the left branch of the dendrogram in Figure 1(a). Similarly, the right branch of the dendrogram captures the survival differences and the order of merging of the six curves in group 2.

### Effect of settings on EACCD

#### Effect of m

**Figure 2:**
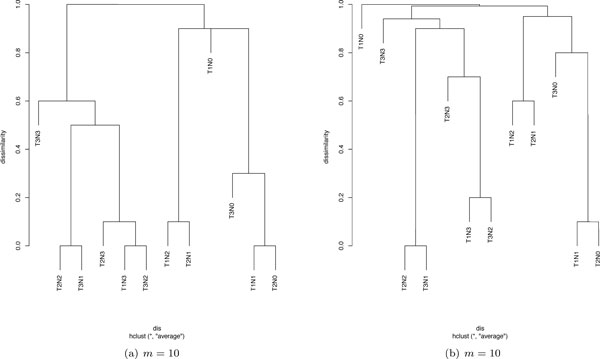
**Dendrograms from the log-rank test, the average linkage, and small *m***.

**Figure 3:**
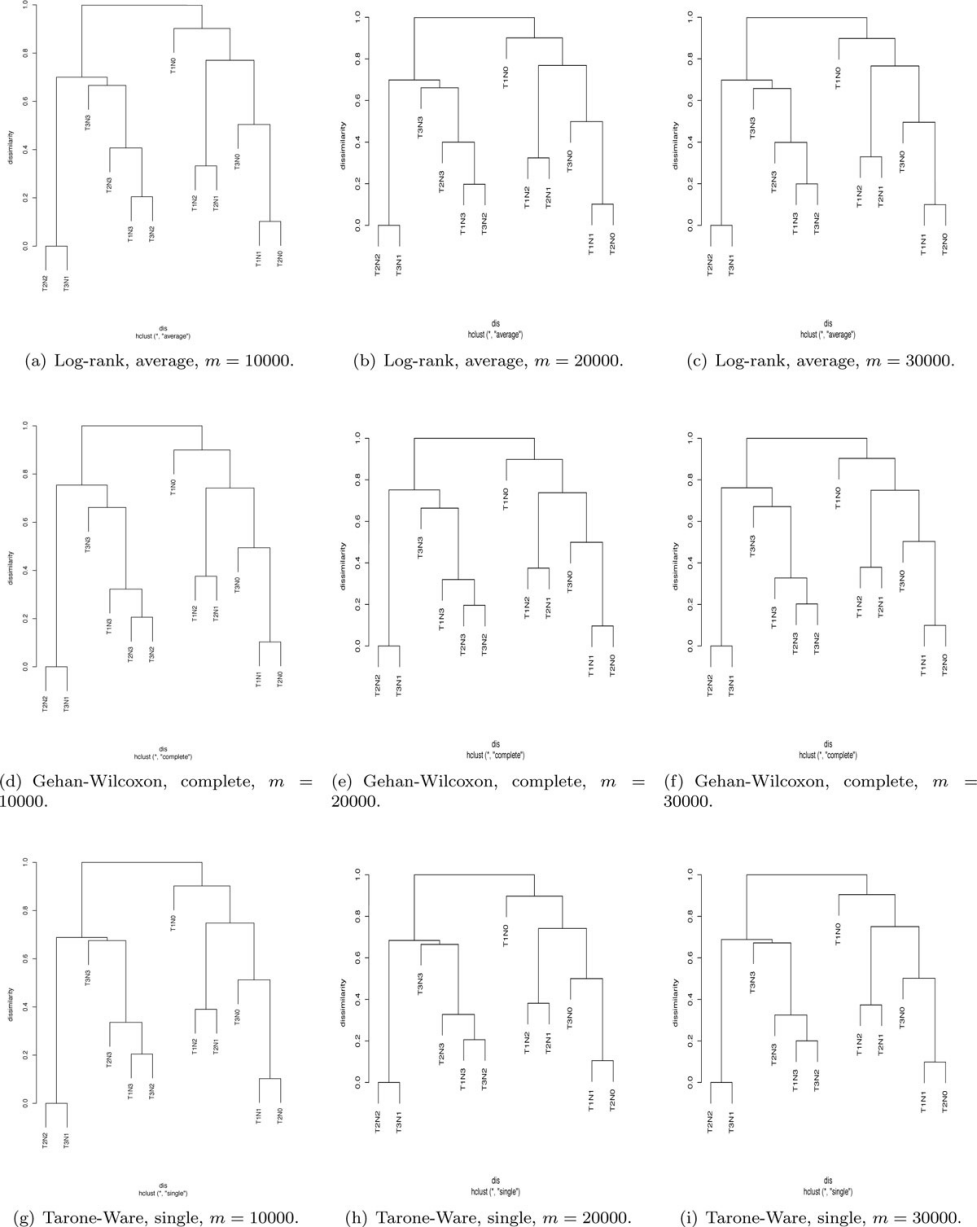
**Dendrograms from large *m *values**.

The learnt dissimilarity "*dis*" in EACCD depends on the values of *m*, which will be convergent when *m *is sufficiently large. If on the the other hand, *m *is small, the dissimilarity is not convergent and can be regarded as a variable. Thus, the resulting dendrograms will not be robust. Specifically, for a small value of *m*, multiple runs of EACCD with the same test and same linkage may produce significantly different dendrograms. This is shown in Figures 2(a) and 2(b). However, when *m *is large, the dendrograms for the same test and same linkage are virtually the same. For example, when *m *= 10000, 20000, 30000, the dendrograms (Figures 3(d), (e), (f)) based on the Gehan-Wilcoxon's test and the complete linkage are similar, and the dendrograms (Figures 3(g), (h), (i)) based on the Tarone-Ware's test and the single linkage are almost identical. Therefore, a large *m *should be used when applying EACCD.

#### Effect of tests and linkage functions

**Figure 4:**
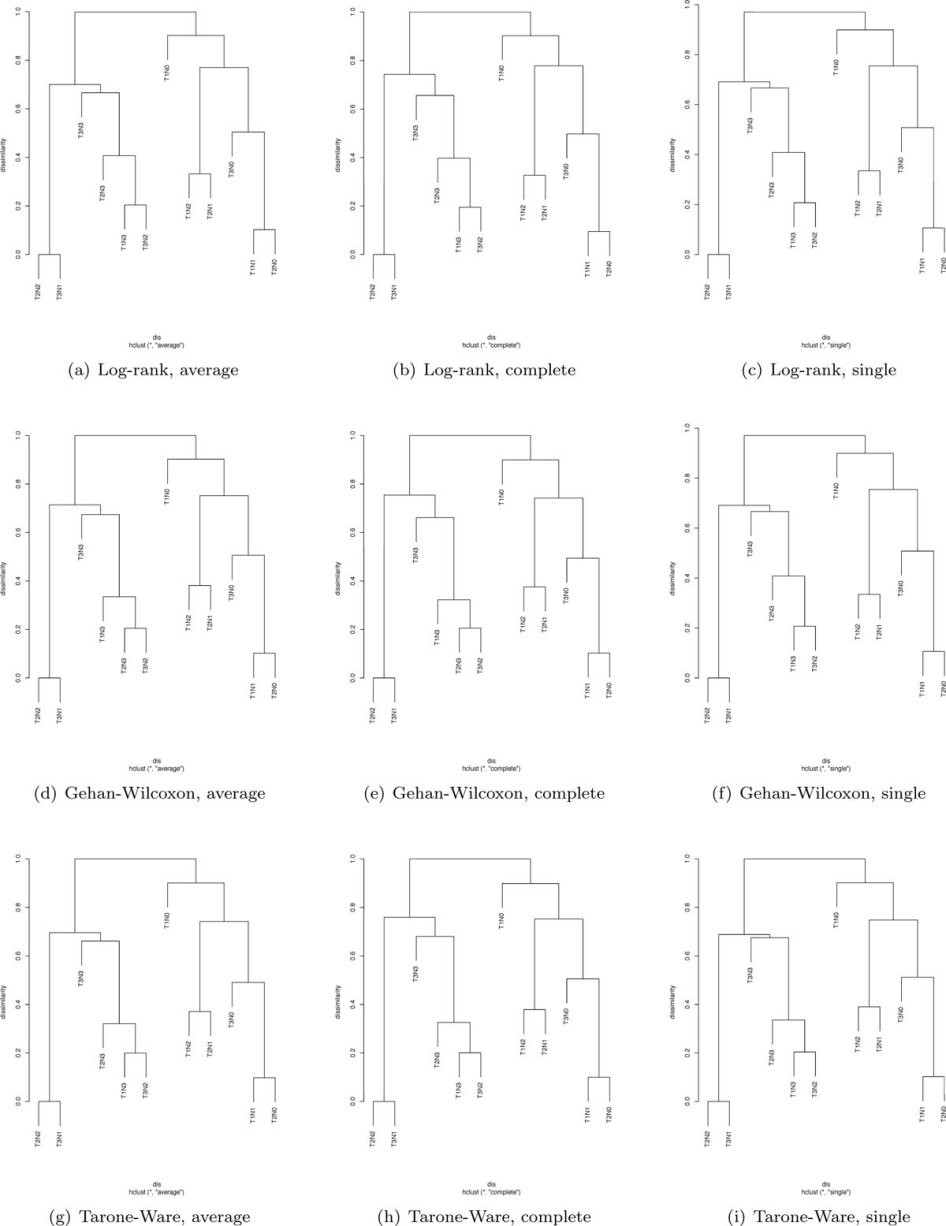
**Dendrograms from *m *= 1000, three tests, and three linkage functions**.

We further examined the effect of statistical tests for large values of *m*. Figure 4 lists nine dendrograms for *m *= 10000, the log-rank test, the Gehan-Wilcoxon's test, the Tarone and Ware's test, the average linkage, the complete linkage, and the single linkage. There were two observations, drawn by visualizing the figure horizontally and vertically. First, for a given test, the dendrograms based on different linkage functions exhibit the same merging pattern, but merging or fusion can occur at significantly different dissimilarity values. For example, with the log-rank test, the dendrogram from the average linkage has the same shape and merging pattern as the dendrogram from the complete linkage. For the average linkage, *T*2*N*2 + *T*3*N*1 is merged with *T*1*N*3 + *T*3*N*2 + *T*2*N*3 + *T*3*N*3 at the dissimilarity of 0.76. But that fusion occurs at the dissimilarity of 0.79 for the complete linkage. Second, for a given linkage, the dendrograms derived from different tests are virtually the same, which indicates that for a given linkage, test statistics have minimal influence on the dendrogram. For instance, Figures 4(a), (d), and 4(g) essentially show the same dendrogram for the average linkage and three tests (the log-rank test, the Gehan-Wilcoxon's test, and the Tarone and Ware's test).

In summary, our experiments have shown that a large *m *( such as *m *≥ 10000 ) should be used in EACCD. For a large *m*, different linkage functions can generate different dendrograms. But different statistical tests have minimal or no influence on the dendrogram.

### Comparisons with alternative approaches

#### Approach A_1_

**Figure 5:**
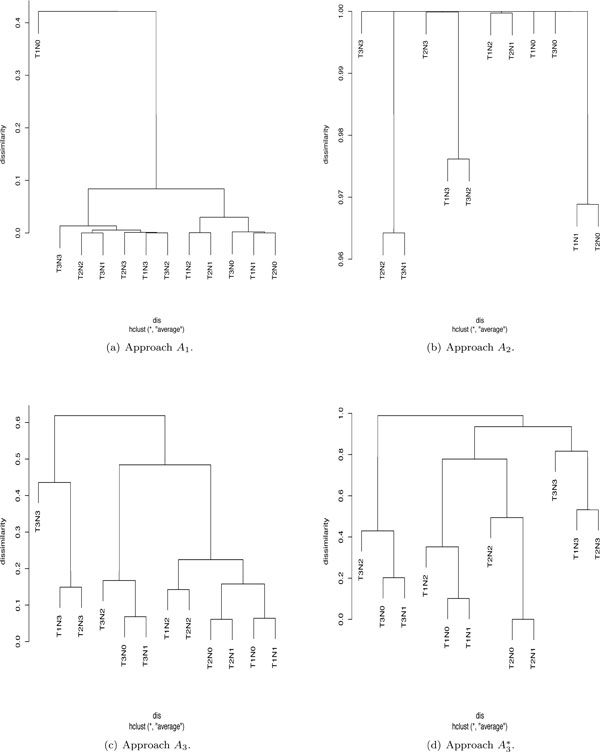
**Dendrograms from various clustering approaches**.

For approach *A*_1_, a hierarchical clustering procedure with the average linkage was applied directly to the breast cancer data. The dissimilarity was determined by the value of the log-rank test statistic. The dendrogram is shown in Figure 5(a). It indicates that *T*1*N*0 becomes a separate group. The reason for this is stated as follows. Consider the set *S *containing all the dissimilarities between one survival function and its "nearest" neighbor, which is identified visually from Figure 1(b). Computation shows that the dissimilarity between *T*1*N*0 and its nearest neighbor *T*1*N*1 is the maximum of *S *and it is nearly 12 times larger than the second largest value in *S*. According to the construction of the dendrogram, *T*1*N*0 is merged with the group of all the other eleven combinations at the last step in the hierarchical clustering procedure.

Note that the combination *T*1*N*0 contains significantly more patients than any other combination (Figure 1(b)). Other experiments showed that if the number of patients in *T*1*N*0 was reduced to a quantity comparable with the number of patients in other combinations, dendrograms from the approach *A*_1 _would have the same shape and merging pattern as in Figure 1(a). This suggests that *A*_1 _is sensitive to the relative size of the combinations.

#### Approach A_2_

The approach *A*_2 _also used a hierarchical clustering procedure with the average linkage to directly cluster the breast cancer data. But in this approach, the dissimilarity was obtained by the p-value from the log-rank test. The dendrogram, shown in Figure 5(b), indicates that the merging steps on the top are not obvious for several combinations. The reason is simply that the dissimilarity 1 − *p *is 1 for most pairs of combinations, due to the rounding effect in computation.

#### Approach A_3_

We employed *A*_3 _to cluster the data by using only *T *and *N*. Survival times were not used with this approach. The corresponding dendrogram is shown in Figure 5(c). Comparing Figure 5(c) with the survival curve plot in Figure 1(b), we can observe that the merging pattern described in the dendrogram at low levels of dissimilarity does not seem reasonable. For instance, the dendrogram indicates that *T*2*N*3 and *T*1*N*3 merge first and then they merge with *T*3*N*3 to form a group without *T*3*N*2, which is not reasonable in light of Figure 1(b). Therefore the traditional clustering procedure using *T *and *N *does not work here. The reason might be that *T *and *N *together could not capture the main information regarding the survival of cancer patients.

The approach *A*_3 _can be modified by incorporating the learning step, as in EACCD. One modification, denoted by $A_{3}^{*}$, is obtained by replacing *dis*_0 _in the first step of EACCD by $dis_{A_{3}}^{s}$ and then following steps 2 and 3 in EACCD with the average linkage. Figure 5(d) shows the dendrogram (*m *= 10000), which again presents unreasonable grouping assignments.

#### Approach A_4_

We ran the PAM algorithm to directly partition the breast cancer data (combinations) for the number of clusters set at each of the following figures: 2, 3, 4, 5, 6, 7, 8, 9, 10, 11. And we obtained the corresponding partition by cutting off the dendrogram in Figure 1(a). Comparisons showed that the results from the PAM and EACCD were the same except for the case where the number of clusters was 4. Table 2 lists the partition results for four clusters from both methods, where a higher group number means a smaller survival in the group. Comparing the table with Figure 1(b), we see that the four clusters from EACCD are reasonable. However, groups 2 and 3 from the PAM show a separation of *T*2*N*1 from *T*1*N*2, which should be placed into the same group as indicated by the survival plot (Figure 1(b)). Therefore, partition of the data from EACCD is more consistent with the survival curves than that from the PAM.

**Table 2:** Partition results for four clusters of SEER breast cancer data from 1990-2000.

	EACCD	PAM
Group 1	T1N0	T1N0
Group 2	T1N1, T2N0, T3N0	T1N1, T2N0, T3N0, T2N1
Group 3	T1N2, T2N1	T1N2, T2N2, T3N1
Group 4	T1N3, T2N2, T2N3, T3N1, T3N2, T3N3	T1N3, T2N3, T3N2, T3N3

In summary, the results of these comparisons have shown that 1) if the step for learning dissimilarity is omitted in EACCD, then the resulting approaches can have a degraded performance, 2) if survival times are not taken into account, then clustering based on prognostic factors will likely generate misleading dendrograms, and 3) direct applications of partitioning techniques to the data can lead to misleading assignments to clusters.

## Conclusion

This report presents a three pronged analysis of EACCD based on a breast cancer patient dataset. First, we examined whether grouping patients by EACCD was consistent with the "natural" grouping of survival curves derived directly from the data. Second, we investigated the effect of different settings in EACCD. Third, we compared EACCD with other clustering approaches. The results showed that if only the PAM is employed for learning dissimilarity, large values of *m *should be used with EACCD and that dendrograms generated from EACCD with the PAM and a large *m *primarily depend on the linkage functions and not on the statistical tests that are used in the learning step. The results also showed that EACCD can be applied to cancer patient data to obtain meaningful dendrograms.

## Competing interests

The authors declare that they have no competing interests.

## Authors' contributions

RQ conceived the study and carried out the experiments. DW conceived the study and carried out the experiments. LS participated in the design of the study. DH prepared the data set, examined the dendrograms, and participated in the design of the study. AS prepared the data set, examined the dendrograms, and participated in the design of the study. EX participated in the experiments. XL conceived the study. DC conceived, designed and guided the study, and wrote the manuscript. All authors have read and approved the final manuscript.

## Declarations

The publication costs for this article were funded by the corresponding author.

This article has been published as part of *BMC Systems Biology *Volume 7 Supplement 4, 2013: Selected articles from the IEEE International Conference on Bioinformatics and Biomedicine 2012: Systems Biology. The full contents of the supplement are available online at http://www.biomedcentral.com/bmcsystbiol/supplements/7/S4.
